# Prevalence norms for 40,777 Catalan words: An online megastudy of vocabulary size

**DOI:** 10.3758/s13428-022-01959-5

**Published:** 2022-09-09

**Authors:** Marc Guasch, Roger Boada, Jon Andoni Duñabeitia, Pilar Ferré

**Affiliations:** 1 Department of Psychology and CRAMC, Universitat Rovira i Virgili, Tarragona, Spain; 2 Centro de Investigación Nebrija en Cognición, Universidad Antonio de Nebrija, Madrid, Spain; 3 Department of Languages and Culture, The Arctic University of Norway, Tromsø, Norway

**Keywords:** Vocabulary knowledge, Word prevalence, Crowdsourcing megastudy, Catalan norms

## Abstract

In this study, we present word prevalence data (i.e., the number of people who know a given word) for 40,777 Catalan words. An online massive visual lexical decision task involving more than 200,000 native speakers of this language was carried out. The characteristics of the participants as well as those of the words which mostly influence word knowledge were examined. Regarding the participants, the analysis of the data revealed that their age was the main factor influencing vocabulary size, followed by their educational level and other variables such as the number of languages spoken and their level of proficiency in Catalan. Concerning the words, by far the most determining factor was lexical frequency, with a minor influence of both length and the size of the orthographic neighborhood. These data mainly agree with those reported in other languages in which the same variables have been analyzed (Dutch, English, and Spanish, thus far). Therefore, the list is increased with Catalan, a language which, due to its use in an essentially bilingual context, is of special interest to researchers interested in the field of bilingualism and second language acquisition.

Mega-studies are large-scale experiments in which thousands of people participate. They have become increasingly popular in psycholinguistics over the course of the last decade, where they have been mostly focused on visual word recognition and have relied on the lexical decision task (e.g., Balota et al., 2007; Ferrand et al., 2010; Keuleers et al., 2010). Such studies were initially conducted in the laboratory, but new approaches have recently emerged, the so-called crowdsourced mega-studies. These involve collecting data from a large number of participants and words outside the laboratory, commonly using online platforms (Keuleers et al., 2015). One of the advantages of such an approach is its reduced cost in comparison to laboratory studies. The other main advantage is that it allows researchers to collect a high number of observations for each word, coming from more heterogeneous groups of language users than laboratory studies. This provides more reliable information on the vocabulary known by the speakers (see Keuleers & Balota, 2015, for an overview). In this paper, we present a Catalan crowdsourcing lexical decision mega-study. Catalan is a Romance language spoken by approximately 10 million people (Escolano, 2021). It is a co-official language in Catalonia, Valencia, and the Balearic Islands together with Spanish. It is also the official language of Andorra, co-official along with Italian in the Italian city of Alghero, and is a language traditionally spoken in the French department of Pyrénées-Orientales. In this work, we address two issues. Firstly, we present word prevalence norms in Catalan based on visual word recognition. Secondly, we examine the contribution of several variables related with the speakers, as well as with the words, to Catalan vocabulary size. In what follows, we develop these issues in more detail.

Word prevalence has been recently proposed as a new measure to capture differences between speakers in word knowledge (Keuleers et al., 2015). It is defined as the percentage of people who indicate that they know a word in lexical decision tasks. Traditionally, word frequency has been one of the variables used as a marker of word difficulty. In fact, it is one of the variables which most influence word recognition, as revealed in many studies that have used the lexical decision task (see Brysbaert et al., 2011). The common finding is that high-frequency words are responded to faster than low-frequency words. Brysbaert et al. (2019), however, noted recently that word frequency is not synonymous with word knowledge. This is particularly true for low-frequency words, among which there are words known by many speakers (‘exclamatory’), while others are mostly unknown by the majority of speakers (‘hypha’).

Keuleers et al. (2015) proposed word prevalence as a better estimate of word knowledge than word frequency. In their seminal study, these authors provided normative data on word prevalence for Dutch, by presenting lists of words and nonwords to large groups of participants and asking them to decide which of those string of letters were real Dutch words. Since then, word prevalence norms have been collected in English (Brysbaert et al., 2019) and Spanish (Aguasvivas et al., 2018). These studies have shown that, although they are correlated, word prevalence and word frequency are not equivalent measures. Keuleers et al. (2015) pointed out that the difference is particularly evident in low-frequency ranges, where prevalence (but not frequency) discriminates between words. Therefore, prevalence is a more refined measure than frequency in the aforementioned cases. Some types of words that exemplify this discrepancy between measures (i.e., low-frequency words which are well known by the speakers) are words which refer to objects from daily life, utensils, loan words, words mainly used during childhood, and some compound and derived words (Brysbaert et al., 2019).

A great interest of this line of research has been to elucidate whether word prevalence contributes to visual word recognition and, if it does, to examine the extent to which this predictive capacity is independent from the effects of word frequency. To address this issue, researchers have used regression analyses where several variables are included as predictors on reaction times obtained from previously published studies using the lexical decision task. Concretely, Brysbaert et al. (2016a) and Keuleers et al. (2015) examined the predictive capacity of word prevalence on reaction times gathered from the Dutch Lexicon Project (Keuleers et al., 2010). Brysbaert et al. (2019) used the same approach taking the reaction time data from the English Lexicon Project (Balota et al., 2007). The results of these studies show that word prevalence explains an additional proportion of variance of reaction times, once the other relevant variables (including frequency) have been considered. Concretely, word prevalence has been shown to explain an additional 6% of variance in Dutch lexical decision times (Brysbaert et al., 2016a) and an additional 3.6% of variance in English lexical decision times (Brysbaert et al., 2019). The effects, albeit smaller, are very similar for word naming (Brysbaert et al., 2019). Interestingly, Keuleers et al. (2015) showed that including the interaction between frequency and prevalence in the regression model resulted in a very small effect size for the interaction term, which led them to conclude that the effects of both variables are additive. Therefore, word prevalence and word frequency are not equivalent, rather complementary, and each of them has a unique contribution on reaction times in word recognition tasks.

In this paper, we present word prevalence norms for Catalan, obtained in a crowdsourced megastudy based on a visual lexical decision task. Following the approach used in previous research (Aguasvivas et al., 2020; Brysbaert et al., 2019; Keuleers et al., 2015), we have focused on native speakers of Catalan. We also present two series of analysis. In the first set of analyses, we examine the variables of the speakers that most influence vocabulary size. In the second set of analyses, we examine the relationship between the prevalence value of each word and several properties of words that are known to affect their recognition. A few of the above studies have examined the role of some of those variables. In them, vocabulary size is computed as the difference between the percentage of hits (correctly accepted words) and the percentage of false alarms (incorrectly accepted nonwords). This line of research has revealed that vocabulary size increases with age, education level (Brysbaert et al., 2016b; Keuleers et al., 2015) and the number of known foreign languages (Keuleers et al., 2015). Furthermore, vocabulary size is slightly larger in males than in females (Keuleers et al., 2015). It has also revealed that word frequency is the most prominent variable in predicting the average accuracy of a word in a lexical decision task, followed by length (the longer the more accurate), and orthographic neighborhood (the smaller the more accurate; Aguasvivas et al., 2020). The influence of those variables is investigated here, too.

In sum, the present article introduces the word prevalence measure for Catalan and examines the role of several variables related to the speakers and to the words in Catalan vocabulary size.

## Method

### Data collection and participants

**Fig. 1:**
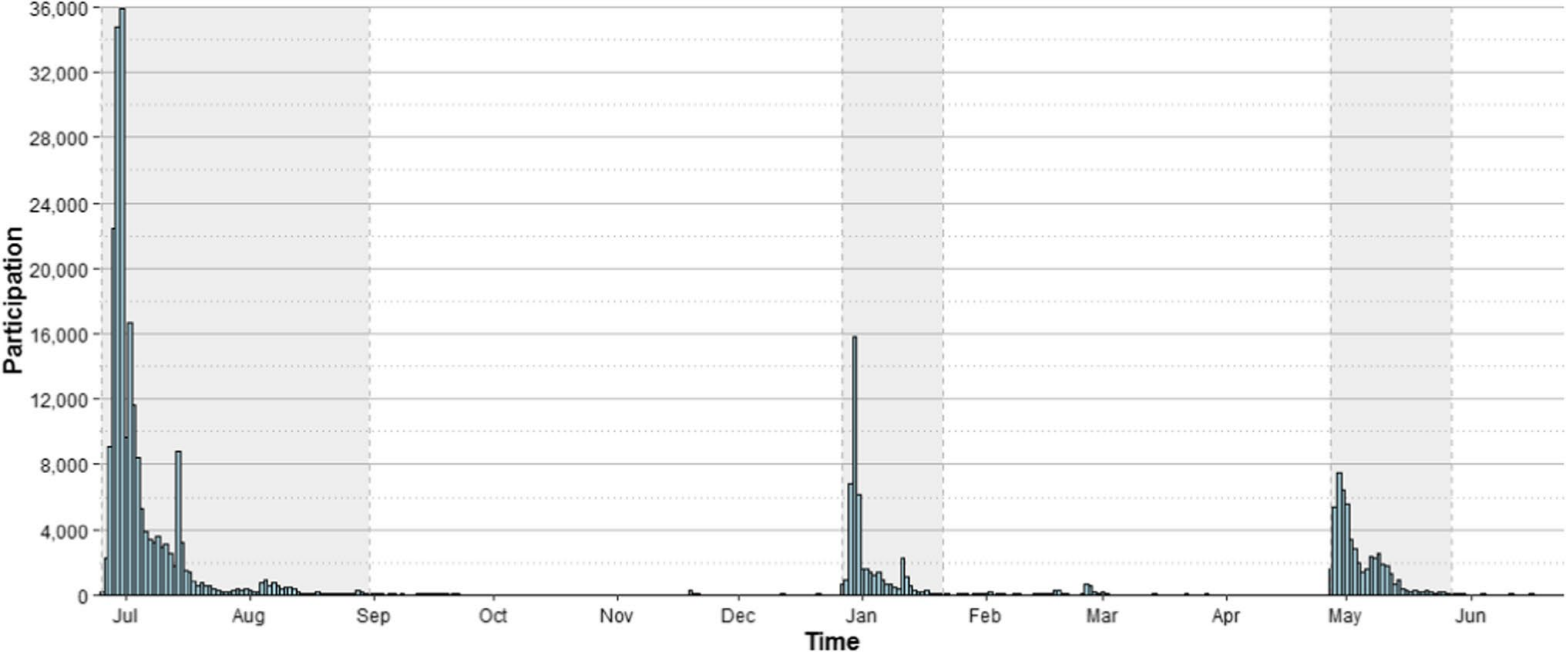
Day-to-day evolution of participation over time. The *shaded areas* mark the periods in which 66, 14, and 17% of the data were collected, respectively

Data were collected online using a web platform over a period of 1 year, starting on June 25, 2019, until June 24, 2020. In total, 319,221 records were collected. Figure 1 shows the day-to-day evolution of participation over time. More than 50% of the data were collected during the first 2 weeks. About 210,000 records were collected during the first wave (the first shaded area of the graph). Two subsequent waves resulted in 45,000 and 54,000 additional records, respectively (the two following shaded areas).

The web platform was disseminated through different social networks and through interviews on radio broadcasts, newspapers, and web portals. Contrary to our expectation, only about 16% of the participants reached the website through social media links, so word of mouth was the main method of dissemination.

**Fig. 2:**
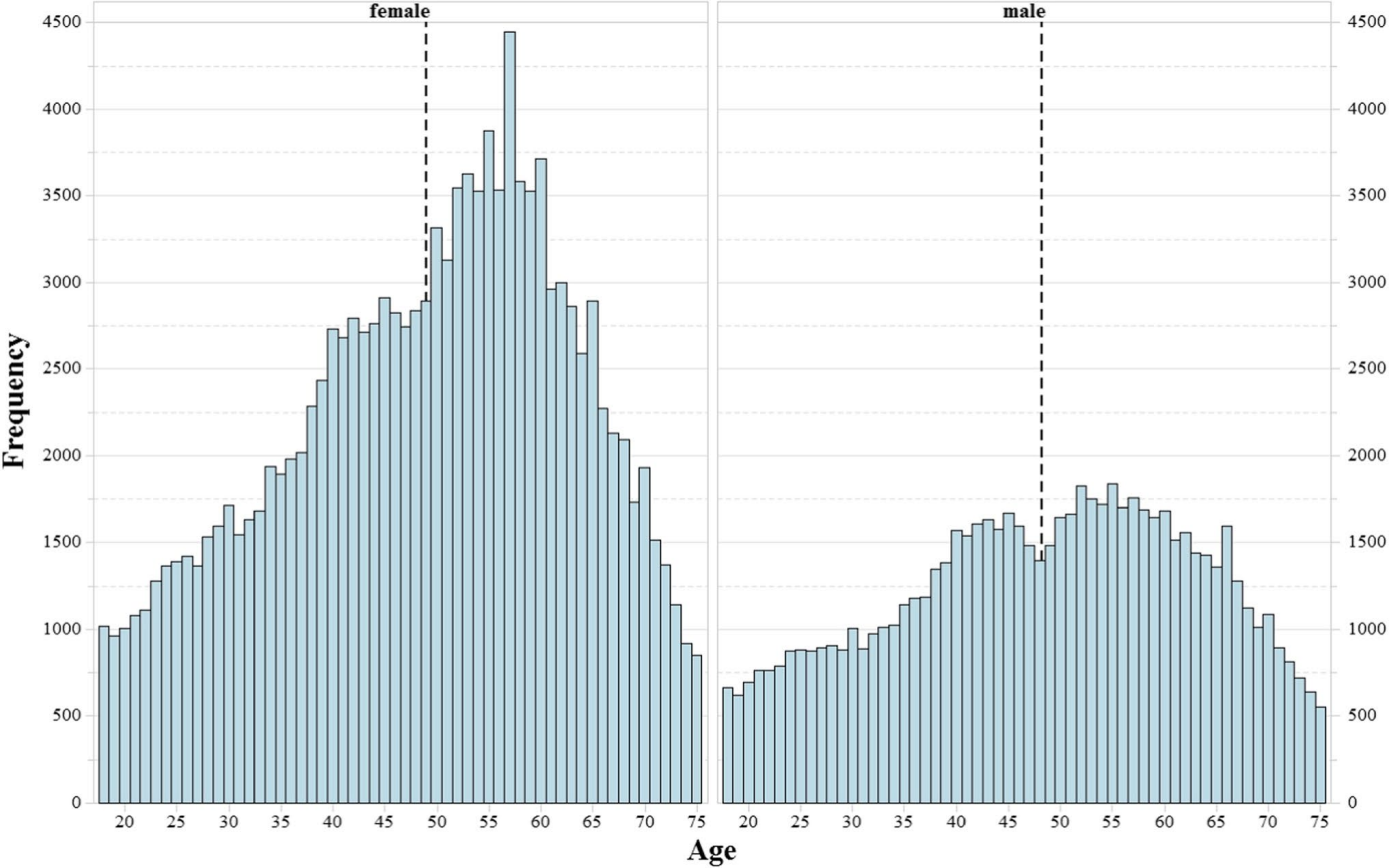
Histograms of records by gender through the different age ranges. The *dashed lines* mark the average age of the group

The final dataset analyzed in the study included 204,645 records (see the data trimming section below), which roughly corresponded to 181,920 different participants^1^. Among the final dataset, 64.66% records were performed by females and 35.34% by males, with a mean age of 48.74 years (SD = 14.23; range, 18–75). Figure 2 shows the distribution of records by gender through the different age ranges.

According to the information extracted from a sociodemographic survey carried out at the beginning of the task (see the Procedure section), participants were mostly born in Catalan-speaking regions (98.89%), and they were exposed to Catalan at a mean age of 0.07 years (SD = 0.38). Catalan was the native language of 90.89% of the participants’ mothers and of 90.76% of the participants’ fathers (of note, both parents of 81.65% of the participants had Catalan as the native language). Participants also reported that they had a mean Catalan proficiency of 6.05 (SD = 1.19) in a 1 (“none”) to 7 (“native”) scale, that they were exposed to Catalan 82.46% of the time on a day-to-day basis (SD = 19.41), and that they spoke 2.94 languages on average, including Catalan (SD = 0.97). Moreover, 69.63% of the participants had at least some university education, while only 3.07% of them had only primary education or no education at all (see Table 1).

**Table 1:** Descriptive statistics for the participants’ variables

Variable	Mean	Median	SD	Range
Age	48.74	50.00	14.23	[18–75]
Education level	-	4	-	[1–5]
Number of languages	2.94	3.00	0.97	[1–7]
Catalan proficiency	-	3	-	[1–3]
Catalan exposure	82.46	90.00	19.41	[0–100]
Score	66.43	67.46	10.80	[36.11–96.43]

Regarding the type of device employed to carry out the task, a touch device was used in 91.91% of the cases (in comparison to a keyboard device). This contrasts with the study by Keuleers et al. (2015; data collected in 2013), where only 27.37% of records were obtained from touch devices. These data give us a hint on the direction to follow in future massive online experiments.

### Materials

To select the words used in the study, we initially relied on the dictionary of the Catalan language of the *Institut d'Estudis Catalans* (2007). This dictionary contains almost 70,000 entries. Starting from a dictionary has the advantage of not including conjugated verb forms or inflections of nouns and adjectives, but it has the disadvantage of including many non-frequent or very specific technical domain words. For this reason, this initial list was cross-referenced with the words included in SUBTLEX-CAT, a subtitle-based lexical frequency database in Catalan (Boada et al., 2020). Words from the dictionary that did not appear in SUBTLEX-CAT were removed, as well as words shorter than two letters or longer than 12 letters. The final list contained 40,777 words.

A large amount of nonwords had to be generated for the purposes of the lexical decision task (i.e., the “no” responses). An ad hoc version of Wuggy (Keuleers & Brysbaert, 2010) was used to this end. Wuggy did not initially support the Catalan language, but the open-source nature of this tool allowed us to construct a version for Catalan by feeding Wuggy with the frequency and the transcription of the syllabic structure of the words included in SUBTLEX-CAT (about 200,000 words). The generated list of nonwords was checked against the list of Catalan words to verify that none of them was an existing word. In addition, we also checked that none of the nonwords was a Spanish (SUBTLEX-ESP; Cuetos et al., 2012) or English (SUBTLEX-UK; van Heuven et al., 2014) word. A total of 30,243 nonwords were used in our study. To assess the suitability of the generated nonwords, we tested the discrimination probability of nonwords with respect to words based on superficial differences between them. For this purpose, we used the LD1NN algorithm (Keuleers & Brysbaert, 2011). This algorithm classifies a novel item in one of two groups (e.g., words or nonwords) by comparing it with the most similar items already seen. The criterion of similarity between items used by the algorithm is based on the edit distance (i.e., the fewest number of changes to convert one string into another). Thus, a result above chance using this procedure indicates a bias in the elaboration of the nonwords that would allow their detection without any previous knowledge of Catalan. In our study, each participant was to respond to 120 stimuli with a word-to-nonword ratio of 70/30 (i.e.: 84 words and 36 nonwords). The reason of such disproportion between words and nonwords is that many low-frequency words are assumed to act as nonwords for most participants. Hence, if we used a 50/50 ratio, this would mean a larger number of "no" responses than "yes" responses for most participants (Keuleers et al., 2015). However, even if words and nonwords were perfectly matched in their form, any sample of 120 items with an unequal ratio between groups would be likely to contain biases. Moreover, even if a list of words in one language were to be compared with another list of words in the same language, it might well be the case that, by chance, the words of one list were significantly more alike than the words of the other list. Therefore, as a first step, we ran 500 iterations of the algorithm by selecting different samples of 84 Catalan words each time to act as words, and 36 Catalan words to act as nonwords. The mean result of the 500 iterations was 2.59 (SD = 0.31). This value should be understood as the odds of responding word versus nonword in a lexical decision task. That is, in this case, the algorithm predicted that a word response was 2.59 times more likely to be correct when a word was presented, than when a stimulus labeled as a nonword by the experimenter (but which was in fact a word) was presented. Taking this value as an intrinsic bias due to the sampling and ratio used, we ran 500 additional iterations comparing our Catalan words with our nonwords. The mean result among iterations was now 2.76 (SD = 0.36), which can be considered as a very close value to the result obtained when comparing two groups of existing Catalan words (i.e., 2.59).

Of further note, we also ran various series of 500 similar iterations by using the Catalan words as words, and word lists from other languages such as Spanish, French, English, Basque, or German as nonwords. The odds of responding word versus nonword were 3.10, 3.63, 3.71, 3.85, and 4.70, respectively. As can be seen, the more different a language is from Catalan, the higher the odds. The mean values obtained in the iterations performed show that our nonwords were more similar to Catalan words than, for instance, Spanish words (of note, Spanish has more than 70% of cognate words with Catalan; Green, 1988). In sum, these data confirmed that our nonwords did not contain obvious biases that would allow a lexical decision task to be carried out simply on the basis of superficial characteristics of the stimuli.

### Procedure

**Fig. 3:**
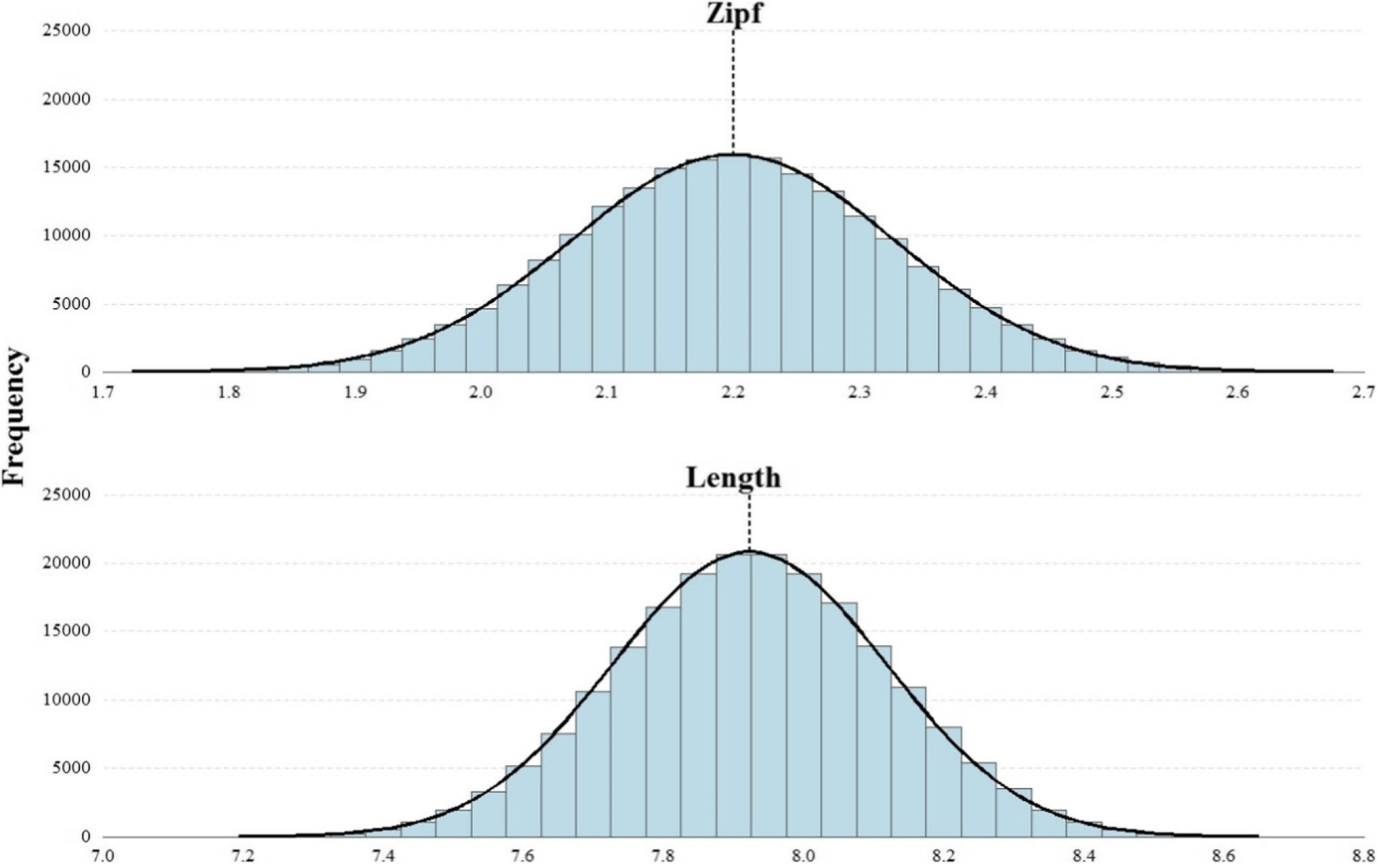
Distribution of the mean frequency of words (Zipf) and the mean item length across sessions, with normal curve. The *dashed lines* mark the respective mean values

The procedure was based closely on that used by Keuleers et al. (2015). Participants had to respond to 120 trials in a proportion of 70% words (i.e., 84), and 30% nonwords (i.e., 36), randomly chosen from the respective pools. No restrictions of any kind were applied when selecting the stimuli, except for a method to avoid large disproportions in the number of occurrences of each item. The method consisted of setting a threshold of occurrences for all words. When a word reached this threshold, it was no longer considered as a candidate. Once all words had reached the threshold or were close to it, the threshold was increased for all items, and they were again candidates to appear in a session. Since the selection of the stimuli in each session was left to chance, we tested after collecting the data if the distribution of the mean frequency of words and the mean item length (considering words and nonwords) in each session were normally distributed (see Fig. 3).

The distribution of word’s Zipf had a mean value of 2.20 (SD = 0.13), ranging from 1.67 to 2.81. The distribution of number of letters had a mean value of 7.92 (SD = 0.20), ranging from 7.03 to 8.81 letters. In both cases, the distribution adopted an appropriate Gaussian distribution.

Data were collected through a web page built from scratch with an html+php+sql framework, and the use of jsPsych library (de Leeuw, 2015). The website was hosted on a server of the Department of Psychology at the Universitat Rovira i Virgili. The task is available online at https://psico.fcep.urv.cat/vocabulari/

The web was divided into several screens. The first screen included a short briefing of the study with links to a contact and a FAQs section, and buttons to share the web through social networks. By clicking the “continue” button, participants agreed to participate anonymously in the study.

Immediately after this, participants were presented with a short sociodemographic survey in which they were asked to provide: their age, gender, place where they were raised, education level, language used by both parents, level of Catalan knowledge on a seven-point scale, age of first contact with Catalan, proportion of time exposed to Catalan in everyday life, and number of spoken languages. Participants had the option to skip this survey and continue with the task, but in that particular case, they were warned that we would not be able to use their data for the study.

Then, the instructions of the task were presented. Participants were told that they were to see 120 letter strings, some of which were Catalan words, and others not (made-up words). They had to decide whether each letter string was a Catalan word or not. In the keyboard device version, they had to press the J key to indicate that they knew the word, ant the F key to indicate that hat they did not know the word. In the touch device version, they had to press a green button placed on the right side of the screen labeled "Yes" to respond that they knew the word, and a red button on the left side of the screen labeled "No" to respond that they did not know the word. They were also warned that answering "Yes" to unknown words would penalize their final score. Participants who used a keyboard device version were asked to place their index fingers on the keys and to press the spacebar to begin the task. For participants who used the touch device version, the "Yes" and "No" answer buttons appeared on the screen, and they had to press "Yes" to start.

The task then started. In each trial, the letter string appeared in the center of the screen, in lowercase, together with the two response buttons in the touch device version, and with reminders of the valid response keys in the keyboard version. After responding, participants did not receive any immediate feedback, and they had no time limit for giving their response. A progress bar appeared at the top of the screen across the entire task. The task took just under 5 min on average.

Once the task was complete, participants received feedback about their global performance. They were informed of their score, the score’s percentile, the percentage of words and nonwords correctly identified and the total number of participants who had taken part in the study up to that moment. The score was computed as the proportion of correctly identified words minus the proportion of nonwords wrongly identified (false alarms; Keuleers et al., 2015). Finally, the link to the definitions of the 84 words presented in the task, obtained from the DIEC2 (*Institut d’Estudis Catalans*, 2007), was provided to each participant, as well as the list of nonwords that he/she incorrectly classified as words. Participants were then given the option to either share their score through the social-media, or to try to improve their score (by running the task again, with other stimuli, without filling in again the sociodemographic questions), or to let another person participate using the same device.

### Data trimming, description, and supplementary materials

A total number of 319,221 records were collected, to which data trimming was applied. Firstly, we discarded any records with technical errors (e.g., character encoding errors, partially saved data, etc.); 0.13% of the total data was removed for that reason. Secondly, we did not consider the records in which the participant decided not to completely fill in the personal data questionnaire (9.20% of the data). Furthermore, as our main interest was to have a reliable measure of the vocabulary known by native speakers of Catalan, we also eliminated those participants who indicated that none of their parents was a native Catalan speaker (15.80% of the data). In addition, given the possibility that there were participants with at least one native Catalan-speaking parent, but who had been raised with another language, only the data of those participants who reported having an age of acquisition of Catalan equal to or younger than 4 years were taken into account (an additional 4.06% of the data was removed for this reason). Data from participants under 18 years were discarded (2.46% of the data), as well as data from those over 75 years (1.77% of the data), given that from that age onwards the number of participants in each age group was very small. Finally, we carried out an outlier removal procedure based on the participants' scores (see the procedure section for the calculation of the score). Outlier detection was carried out using the boxplot method, where we used a criterion of ± 1.5 times the interquartile distance to establish the fences. This was done using the *univOutl* package (D’Orazio, 2021) in R (R Core Team, 2021), on log-transformed scores, and with asymmetric fences to account for skewness in the distribution. This led to the exclusion of 2.45% of the data from the lower part of the distribution (corresponding to records of participants who may not have been paying attention or who were not doing the task correctly), and 0.03% of the data from the upper part (which might correspond to participants who cheated). As a result of the overall data trimming, the final dataset included 204,645 records from 181,920 participants.

**Fig. 4:**
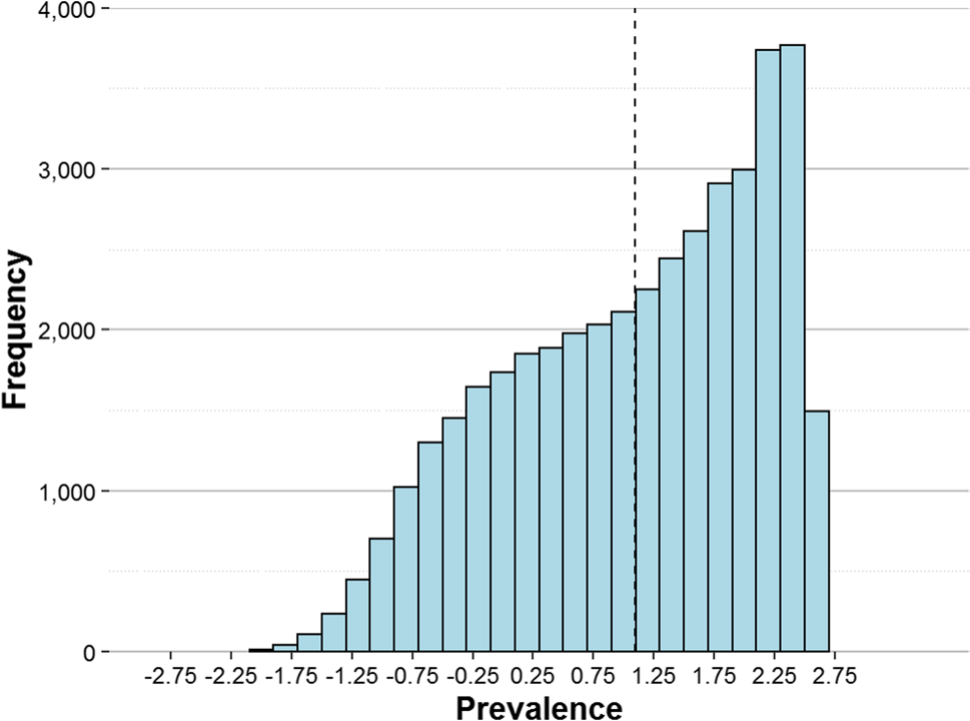
Histogram of word prevalence. The *dashed line* marks the mean prevalence score

The percentage of knowledge of each word was calculated from the final dataset. Each of the 40,777 words had 421.57 observations on average (SD = 79.28; range [350–2479])^2^. The mean word knowledge (i.e., the percentage of participants who knew a particular word) of the entire set was 77.14 (SD = 26.50). Additionally, we computed the prevalence value of each item as described in Brysbaert et al. (2019). This is a transformation of the percentage of knowledge that converts it into a score ranging from – 2.576 to + 2.576. The main advantage of such conversion is that it differentiates better between words with a high percentage of knowledge than the raw score. A prevalence of zero for a particular word indicates that it is known by 50% of participants. Figure 4 shows the distribution of word prevalence.

**Fig. 5:**
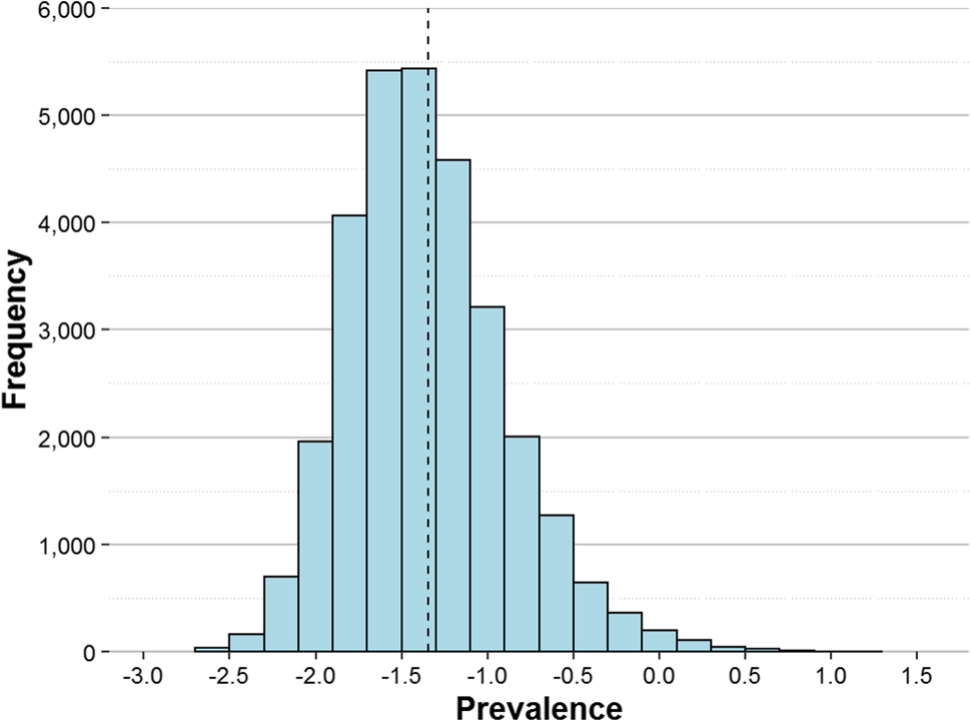
Histogram of nonword prevalence. The *dashed line* marks the mean prevalence score

On the other hand, the mean number of observations for the 30,243 nonwords was 243.60 (SD = 59.74; range [182–2289]). The nonwords had a mean false-alarm rate (nonwords that have been identified as words) of 10.74 (SD = 9.82). We also applied the prevalence transformation to false-alarm rates because it can contribute to discriminating between nonwords in the low range. This information can be most useful when it comes to selecting nonwords in the Catalan language for lexical decision tasks, thus also allowing the nonwords’ difficulty to be graded. Figure 5 shows the distribution of nonword prevalence. As can be seen, there are very few nonwords that fooled more than half of the participants (mainly pseudohomophones), but there is an even distribution of values with a prevalence value below zero.

The norms are available at 10.6084/m9.figshare.16622536.v3, where several files can be found. One folder contains the trimmed raw data together with an R script for processing it. The other folder contains the processed data in .xlsx and .txt format. The “items” files contain the information about the words, including an id for each word, the list of words, the percentage of people that knows each word, the prevalence score of each word and its number of observations. The values of a set of lexical variables, extracted from SUBTLEX-CAT (Boada et al., 2020), are also included: length, Coltheart's N, OLD20, relative lexical frequency, Zipf, relative contextual diversity, and the logarithm of the contextual diversity.

The “items” files also contain information about the nonwords: the list of nonwords and the same information as that provided for words, except for the variables related to frequency of use. Note that the percentage of knowledge for nonwords needs to be interpreted as the percentage of false alarms, and the same holds for prevalence scores. Coltheart's N and OLD20 for the nonwords were computed from scratch using the words in SUBTLEX-CAT and the *vwr* package (Keuleers, 2013) in R (R Core Team, 2021).

The “sessions” files contain the information about the participants, which was obtained from the sociodemographic questionnaire (e.g., age, gender, education level, etc.). They also include the score obtained by each participant in the task, the percentage of correctly identified words, and the percentage of incorrectly identified nonwords (false alarms).

## Results and discussion

We carried out a series of analyses to characterize the dataset. These analyses are divided into two sections, one focused on participants and the variables that affect their vocabulary size, and the other focused on words and the variables that influence word knowledge.

### What can we learn from participants?

We ran a multiple regression analysis to examine the sociodemographic variables that can influence vocabulary size. The dependent variable was the score of each participant in the task, and the predictors were some of the variables collected in the questionnaire. We did not include as predictors the variables that were already used for the final selection of participants (i.e., the language used by both parents, and the age of first contact with the Catalan language). We also discarded the information about the place where the participants were raised, because only 1.12% of the participants grew up in a place where Catalan was not spoken. Therefore, the predictors included in the model were age (log-transformed to correct for the non-linear relationship with vocabulary size), number of spoken languages, proportion of time exposed to Catalan in everyday life, gender, self-perceived level of Catalan knowledge, and level of education.

Regarding the level of Catalan knowledge (i.e., Catalan proficiency), it was initially assessed on a seven-point scale ranging from no level of Catalan to a native level. However, the variable was recoded into three levels because some levels in the original scale had a very small number of observations. The first level (“low level”) included participants with no knowledge of Catalan at all; those with a very low level, and those with a low level of knowledge in the original scale (1.19% of the participants). The second level (“medium level”) corresponded to participants who indicated in the original scale that they had a medium knowledge of Catalan (12.19% of the participants). Finally, the third level (“high level”) included the participants who reported to have either a high, a very high or a native-like level of Catalan in the original scale (86.62% of the participants).

Concerning education, we asked in the questionnaire for the highest level of education attained or in progress. The responses were classified for the purposes of the regression analysis as: 1 = primary education or no formal education (3.07% of the data, but only 0.16% of the data corresponded to no formal education), 2 = secondary education (4.24% of the data), 3 = all the educational levels between secondary school and university entrance (including both baccalaureate and professional training; 23.06% of the data), 4 = university degree (48.65% of the data), and 5 = master or doctoral studies (20.98% of the data). Descriptive statistics for these variables can be found in Table 1, and the correlation between them can be seen in Table 2.

**Table 2:** Correlations between participants’ variables

Variable	log(Age)	Gender	Education level	Number of languages	Catalan proficiency	Catalan exposure	Score
log(Age)	1.00	.03	– .27	– .14	– .25	.10	.29
Gender	.03	1.00	.03	– .06	.01	.12	– 0.6
Education level	– .27	.03	1.00	.32	.26	– .06	.10
Number of languages	– .14	– .06	.32	1.00	.20	– .11	.11
Catalan proficiency	– .25	.01	.26	.20	1.00	.06	.07
Catalan exposure	.10	.12	– .06	– .11	.06	1.00	.04
Score	.29	– .06	.10	.11	.07	.04	1.00

*N* = 204,645. All *p*s < .001. Pearson's correlations except when the ordinal recoded variables (education level and Catalan proficiency) are involved. In these cases, the table shows Spearman's rho correlations. For the point-biserial correlation with gender: male = 0, female = 1

The procedure employed to select the terms of the model was the following: We started including all the factors and then compared the resulting model with the same model without the proportion of time exposed to Catalan in everyday life. We selected this factor because it showed a very small variability in the sample (more than 85% of participants reported being exposed to Catalan between 70% and 100% of the day). It seemed also to be the less relevant variable, to the extent that it was not considered in previous studies (Keuleers et al., 2015; Aguasvivas et al., 2020). The full model accounted for 13.94% of the variance of the dependent variable and had and AIC value of 1,524,028. The model without the proportion of time exposed to Catalan accounted for 13.86% of the variance of the dependent variable and had and AIC value of 1,524,225. The difference in *R*^2^ between both models was significant [*F*(1, 204638) = 199.65, *p* < .001], and the full model had a lower AIC. Therefore, we chose the model with all predictors as the final one. We also examined the predictive capacity of the interactions between variables, by comparing a model including all two-way interactions with our previous model. The 15 possible two-way interactions considered together only added a 0.15% to the variance explained by the model (*R*^2^ = 0.1409), and none of the interactions alone explained more than 0.04% of variance. This indicates that, although we should be aware of these interactions (e.g., participants who self-assess themselves as more proficient in Catalan also have a higher level of education), their influence on the overall pattern of effects is almost negligible.

Table 3 shows the results of the final model [*R*^2^ = 0.1394, *F*(6, 204638) = 5526, *p* < .001]. In what follows, we discuss the effects of each predictor in order of importance, except for the time of exposure to Catalan because this particular predictor explains a very low percentage of the overall variance (0.08%).

**Table 3:** Regression coefficients and analysis of the variance table with the effects of the examined predictors on participants’ scores

Variable	B	SE	β	95% CI	SS	*df*	*F*	*p*	*η*^2^
log(Age)	11.44	0.07	0.35	[11.30, 11.57]	2,706,867	1	26958.24	< .001	0.1134
Education level	1.54	0.03	0.13	[1.49, 1.59]	341,256	1	3398.64	< .001	0.0143
Number of languages	1.14	0.02	0.10	[1.09, 1.18]	220,716	1	2198.16	< .001	0.0092
Catalan proficiency	2.53	0.06	0.09	[2.41, 2.65]	170,433	1	1697.38	< .001	0.0071
Gender	-1.77	0.05	-0.08	[-1.86, -1.68]	143,405	1	1428.20	< .001	0.0060
Catalan exposure	0.02	0.001	0.03	[0.01, 0.02]	20,047	1	199.65	< .001	0.0008
Residuals					20,547,620	204,638			

SE = standard error. CI = confidence interval. SS = sums of squares. *df* = degrees of freedom. *η*^2^ = effect size (eta-squared)

#### Age

**Fig. 6:**
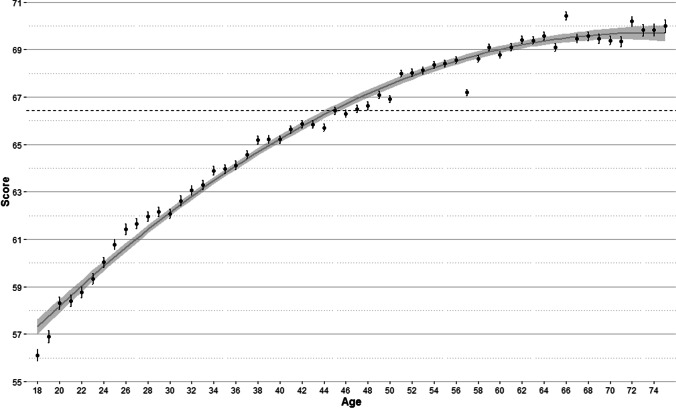
Mean scores by age with standard *error bars*. The *grey line* is a quadratic regression curve with a 95% confidence interval (*shaded area*). The *dashed line* marks the global mean score

Participants’ age was the main predictor of vocabulary size, accounting for 11.34% of the variance. This result is in line with that of Aguasvivas et al. (2020) and Keuleers et al. (2015), who also reported age as the most relevant predictor (note that participant-related variables were not examined in the paper about English word prevalence of Brysbaert et al., 2019). Figure 6 clearly shows that participants’ scores raise as age increases.

The mean score of participants who are 18 years old is around 56%. If we interpret the score as the percentage of words known (Keuleers et al., 2015), this would mean that participants at this age know about 22,000 words. This contrasts with the mean score obtained by 75 years-old participants, almost 70%, which means that they would know more than 28,000 words. It should be noted, however, that the increase in the score with age does not follow a linear but a curvilinear relationship, with the slope of the curve being steeper at younger ages and attenuated at older ages (see Fig. 6). For instance, between the ages of 20 and 30 years the score increases by 3.8 points, while the increase is reduced to 1.7 points between the ages of 40 and 50, and to 0.6 points between the ages of 60 and 70. This pattern (i.e., a decrease in vocabulary growth with age) is very similar to that observed by Keuleers et al. (2015), and Aguasvivas et al. (2020). Importantly, albeit at a slower pace, vocabulary keeps increasing at old ages. The active lifestyle of most old people today, with stimulating leisure activities, may contribute to this fact.

**Fig. 7:**
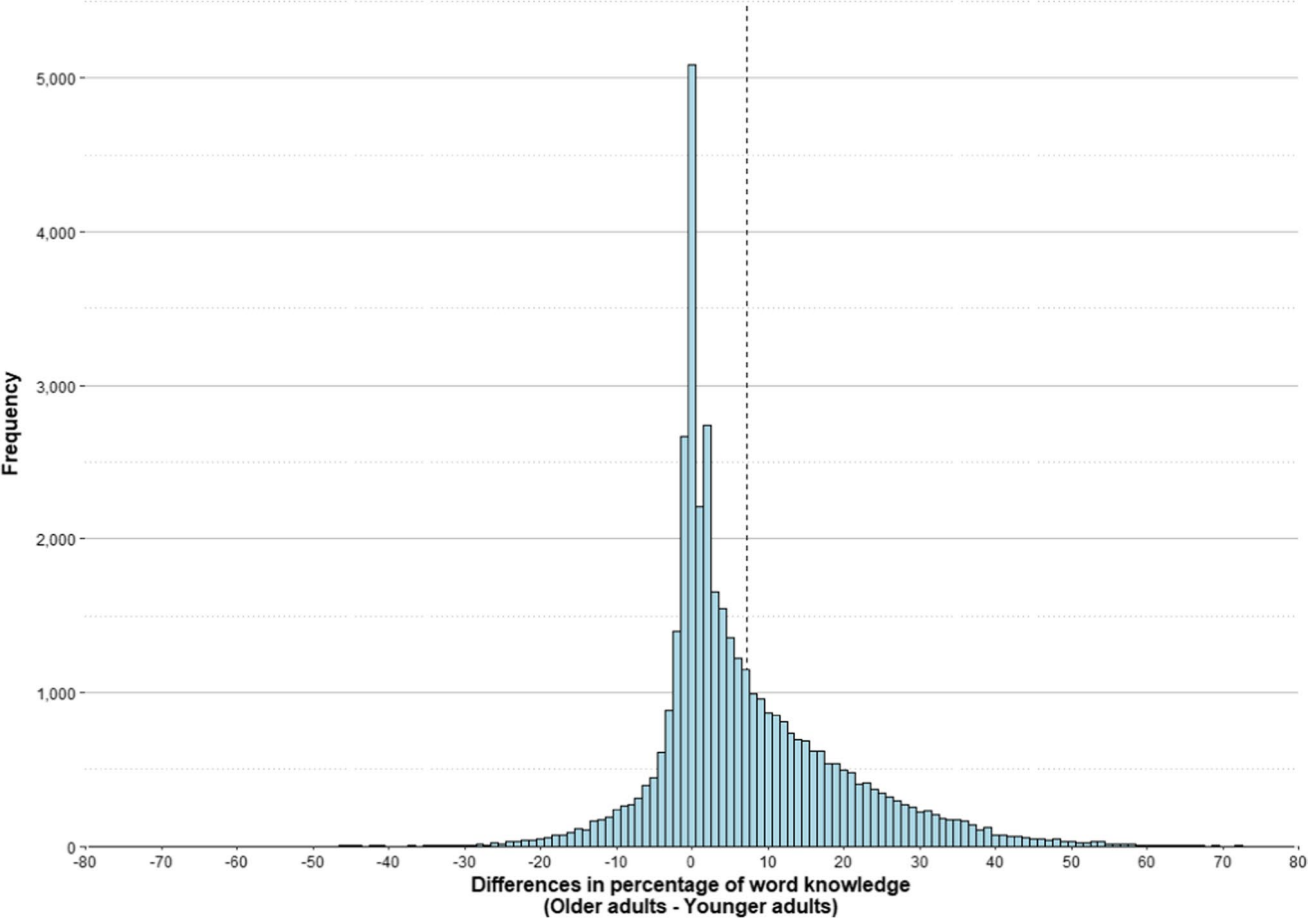
Histogram of differences in percentage of knowledge of words between old adults and young adults. The *dashed line* marks the mean of these differences

We conducted a series of additional analyses to examine in more detail the difference between young and old participants in vocabulary knowledge. We calculated the percentage of knowledge of each word for participants between 18 and 30 years old (young adults), and for participants between 60 and 75 years old (old adults). The correlation between the values was very high *r*(40775) = .90, *p* < .001, indicating that the more familiar a word is to one group, the more familiar is also to the other group. We also computed the difference in word knowledge for each word by subtracting its percentage of knowledge in young adults from its percentage of knowledge in old adults. A positive value for this variable indicates that the word is better known by old adults than by young adults, while a negative value indicates that it is better known by young adults than by old adults. Figure 7 shows the distribution of these differences.

As can be seen in Fig. 7, the distribution is very asymmetric. The majority of words (84.85%) did not differ in percentage of word knowledge between young and old participants by more than a 20% upward or downward. On the other hand, only 0.66% of the words showed a 20% of difference in favor of the youngsters (i.e., young adults knew these words better than old adults), the maximum difference being 52.30%. In contrast, 14.49% of the words showed a difference of more than 20% in favor of the oldest participants (the maximum difference was 83.18%). Thus, it can be said that the elders knew practically all the words known by the youngsters, plus other words. When we looked at the words that showed the greatest differences, we realized that at the top ten of the words better known by young people were words from the school or academic fields (e.g., eukaryote, prokaryote, hyperonym, anaphora). In contrast, at the top ten of the words better known by older people were old-fashioned words or words that have fallen into disuse (e.g., astrakhan, cyclostyle, telex, cinemascope).

#### Education level

**Fig. 8:**
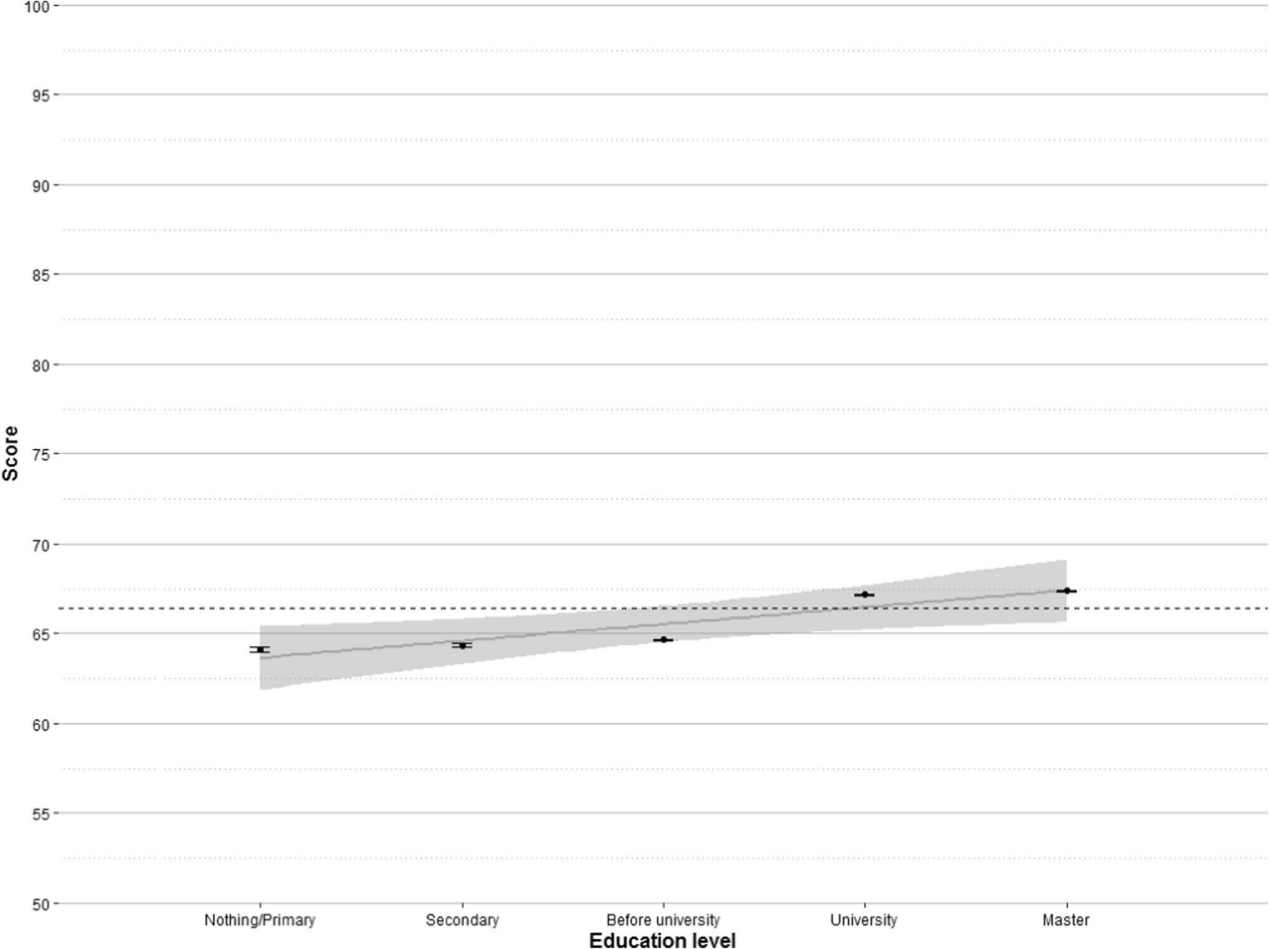
Mean scores by education level with standard error bars. The *grey line* is a regression line with 95% confidence interval (*shaded area*). The *dashed line* marks the mean global score

The second main predictor of participants’ score was education level, with 1.43% of variance explained (see Fig. 8).

Overall, educational level predicts vocabulary size. Participants with no formal education or with only primary education had an average score around 64% (which is equivalent to knowing about 26,000 words), while participants with education level beyond a university degree had an average score over 67% (i.e., more than 27,000 words). However, as can be seen in Fig. 8, the increase does not appear to be linear and constant. Rather, there seems to be a slight gradual increase between primary education (64.11) and university entrance (64.66), and another slight increase between university degree (67.19) and postgraduate studies (67.39). The largest increase was the one observed between the “before university” level (which includes baccalaureate and professional training in the Spanish educational system) and the “university” level (a score increase of 2.53 points; more than 1,000 words). The pattern of findings obtained here, that is, the enhancement in vocabulary size as the educational level increases, is in line with that reported in previous studies (Aguasvivas et al., 2020; Keuleers et al., 2015), although in those cases the effects of education were more gradual. This may have to do with the different levels considered in relation to distinct education national systems.

#### Number of languages

**Fig. 9:**
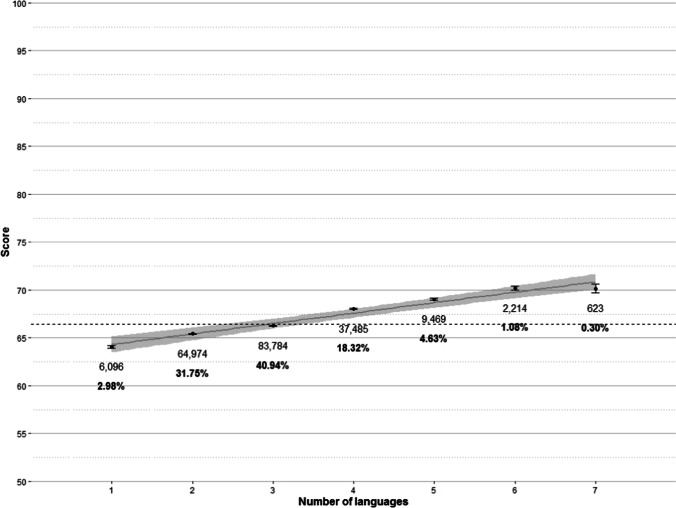
Mean scores by number of languages spoken by the participants, with standard error bars. The *grey line* is a regression line with 95% confidence interval (*shaded area*). The *dashed line* marks the global mean score. *Labels* indicate the number of participants and the percentage of participants (*in bold*) who reported speaking a certain number of languages (from 1 to 7)

We asked for the total number of languages spoken (including the native language) rather than for the number of foreign languages spoken (like Aguasvivas et al., 2020 and Keuleers et al., 2015 did). The reason has to do with the linguistic characteristics of Catalonia. Catalan and Spanish are co-official languages in Catalonia, and they are present in everyday life. Inhabitants of Catalonia are exposed to both languages since an early age. Consequently, most of them are highly proficient Catalan-Spanish bilinguals. Therefore, the most frequent response to the question about the number of languages spoken was three (that typically refer to Catalan, Spanish, and English), and 91% of the responses to this question were clustered around two, three, and four languages. The results of the regression analysis showed that the number of spoken languages explains 0.92% of the variance. Such relationship between number of languages spoken and vocabulary size (which can be observed in Fig. 9), is in line with the results of Keuleers et al. (2015), and Aguasvivas et al. (2020).

As can be seen in Fig. 9, the more languages known, the higher the vocabulary size in the native language. The difference in scores between speaking one language (64.07) and speaking seven languages (70.14) was around six points: a difference of almost 2,500 words. No differences were observed between speaking six and seven languages, although it should be noted that only 0.30% of the sample reported speaking seven languages.

The facilitative effect of number of languages may have different causes. One possibility is that it is an effect of higher education in disguise. That is, participants with a higher educational attainment would know more languages. However, this explanation is unlikely considering the negligible predictive capacity of the interaction between number of languages and education level in the regression analysis (0.02% of variance explained). Another possible reason is the presence of cognate words with the other languages known by the speakers (Aguasvivas et al., 2020; Keuleers et al., 2015). This is a plausible explanation, considering the facilitative role of orthographic similarity (i.e., cognate status) on word learning in a second/foreign language (e.g., Casaponsa et al., 2015; Comesaña et al., 2012). We were not able to examine this possibility in our data because participants were not asked to list the languages they know, but the total number of languages known. Further studies should collect this information and explore the relationship between vocabulary size in the test language and the percentage of cognates with the other languages known by the speakers.

#### Catalan language level

**Fig. 10:**
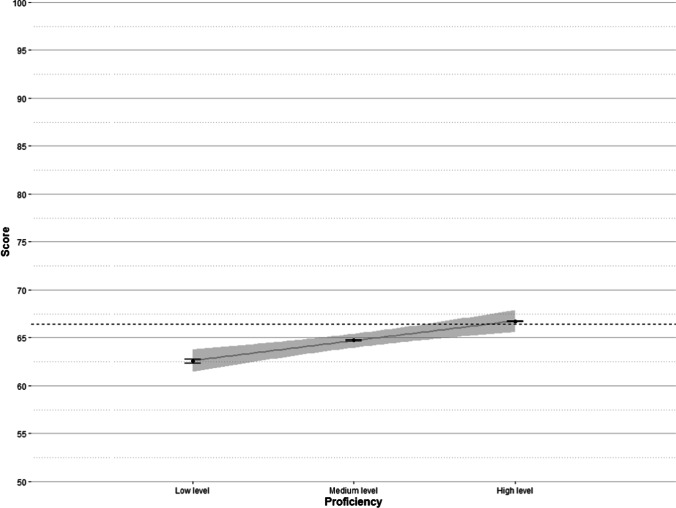
Mean scores by self-reported proficiency with standard error bars. The *grey line* is a regression line with 95% confidence interval (*shaded area*). The *dashed line* marks the global mean score

Our inclusion criteria (participants mostly born in Catalan-speaking regions, who had at least one Catalan native parent, who were exposed to Catalan since birth and who had a high degree of exposure to Catalan in a daily basis) led to a sample of highly proficient Catalan speakers. Despite that, there was some variability in their self-reported proficiency. As indicated above, the initial seven-point scale was recoded into a three-point scale (“low”, “medium”, and “high”). Figure 10 shows the relationship between self-reported proficiency in Catalan and participants’ scores. This variable explained 0.71% of the variance.

Participants who felt that they had a low level of proficiency in Catalan were also those with the lowest scores (a mean score of 62.59), while participants with a self-reported high level of proficiency in Catalan were those with the highest scores (a mean score of 66.72). This means a difference in vocabulary size of more than 1,500 words. The relationship between self-rated proficiency and the score suggests that participants have a realistic idea of their proficiency level in their native language.

#### Gender

**Fig. 11:**
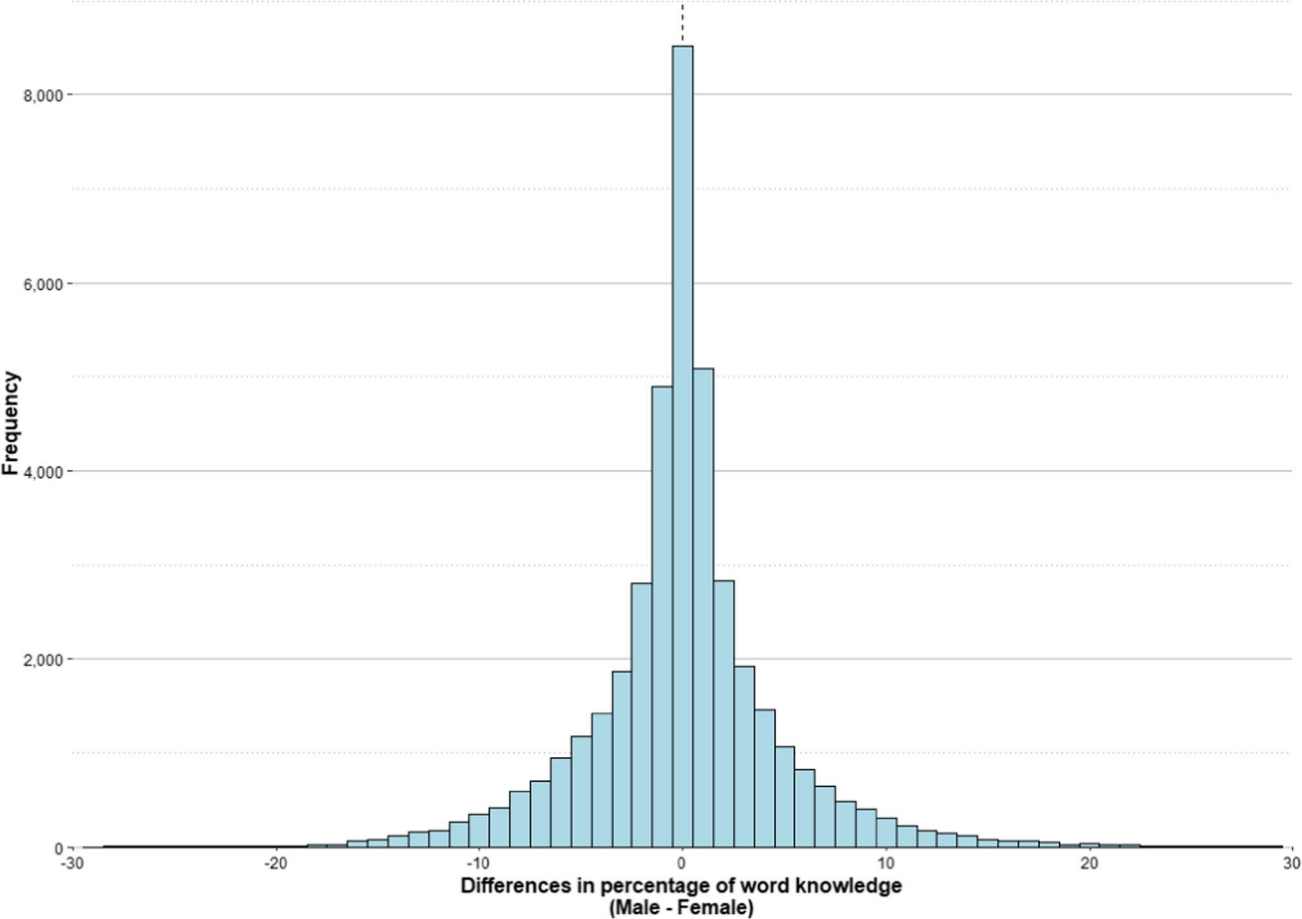
Histogram of the differences in percentage of word knowledge between males and females. The *dashed line* marks the mean of this difference

Gender explained a 0.60% of variance. Males scored 1.46 points higher than females (slightly more than 500 words). Reminiscent of what we did for the age variable, we computed the percentage of knowledge of each word for males and females, and the difference between both ratings (males minus females) for the entire set of words. A positive value for this difference indicates that the word is better known by males than by females, while a negative value indicates the opposite. The correlation between word knowledge of males and females was very high *r*(40775) = .98, *p* < .001. Figure 11 shows the distribution of the differences between males and females in percentage of word knowledge.

In this case, the distribution of the differences was extremely symmetrical, with the mean of the differences being 0.002 (SD = 4.80), and 99.59% of the words differing by no more than 20% in either direction. Only 0.14% of words were better known by females than by males with more than 20% difference (being 35.34 the maximum difference), while 0.28% of words showed the opposite pattern (i.e., males knew these words better than females, being 35.41 the maximum difference). Among the top ten words that were best known by male participants, there were terms referring to materials (e.g., tungsten, ferrite, wolfram). In contrast, the top ten words that were best known by female participants referred to terms related to health (e.g., dysmenorrhea, petechiae), or to fabrics (e.g., organdie, spandex).

The slight superiority for male participants found in this study is in line with the results obtained by Aguasvivas et al. (2020) and Keuleers et al. (2015). Both studies reported a small difference in vocabulary size according to the gender of the participants. Aguasvivas et al. (2020), through an informal exploration of their data, observed that, although the gender effect was observed in all the ages, it seemed to be slightly larger for participants older than 35 years. This makes sense considering the larger differences in education level between women and men in older generations than in younger generations. Nevertheless, it is important to note that whatever the cause of gender differences, they explain a very small effect size. This fact, together with the high correlation between values of word knowledge in males and females and the huge symmetry in the distribution of differences, lead us to consider gender differences in vocabulary as unremarkable (see Aguasvivas et al., 2020, for the same conclusion).

### What can we learn from words?

We examined the relationship between the prevalence value of each word (i.e., the transformation of the percentage of knowledge that converts it into a score ranging from – 2.576 to + 2.576) and the main variables that are known to affect word recognition: word frequency, word length, and similarity between words (Brysbaert et al., 2019). We used the Zipf value (van Heuven et al., 2014) as a measure of word frequency. Word length was computed as number of letters. Regarding the similarity between words, we used the OLD20 value, a measure of orthographic neighborhood density, which is computed as the mean edit distance from a word to its 20 closest orthographic neighbors (Yarkoni et al., 2008). All these data were obtained for the full set of words from SUBTLEX-CAT (Boada et al., 2020). Descriptive statistics for these variables can be found in Table 4, and the correlation between those variables can be seen in Table 5.

**Table 4:** Descriptive statistics for the words’ variables

Variable	Mean	Median	SD	Range
Zipf	2.20	2.07	1.18	[0.56–7.52]
Length	7.92	8.00	2.18	[2–12]
OLD20	2.34	2.20	0.77	[1–6.15]
Prevalence	1.09	1.27	1.06	[– 2.07–2.58]

**Table 5:** Correlations between words’ variables

Variable	Zipf	Length	OLD20	Prevalence
Zipf	1.00	– .27	– .36	.67
Length	– .27	1.00	.77	.16
OLD20	– .36	.77	1.00	– .08
Prevalence	.67	.16	– .08	1.00

*N* = 40,777. All *p*s < .001

We entered Zipf, length, and OLD20 in a multiple regression analysis with prevalence as the dependent variable. We explored the data for possible multicollinearity issues because length and OLD20 are two variables highly correlated. The multicollinearity coefficients were acceptable, with a minimum tolerance of 0.39 and a maximum Variance Inflation Factor of 2.58 (in both cases relative to the OLD20). Therefore, we compared the model with the three predictors with a model without the OLD20 factor. The first model accounted for 59.99% of the variance with an AIC value of 82,911, while the second model accounted for 57.99% of the variance and had an AIC value of 84,893. The difference in R^2^ between both models was significant [*F*(1, 40773) = 2033.33, *p* < .001]. Furthermore, given the interactions observed in previous studies (Aguasvivas et al., 2020) of Zipf with both length and OLD20, we decided to test a model that included these two interactions. In comparison with the model without interactions, this model increased the variance explained to 61.30% and reduced the AIC to 81,559, so this was the final model chosen [*R*^2^ = 0.6130, *F*(5, 40771) = 12920, *p* < .001].

Table 6 shows the results of the final model. The effect of the predictors is explained below, by order of importance.

**Table 6:** Regression coefficients and analysis of the variance table with the effects of the examined predictors on word prevalence

Variable	B	SE	β	95% CI	SS	*df*	*F*	*p*	*η*^2^
Zipf	0.71	0.01	0.79	[0.69, 0.72]	21,356	1	49368.1	< .001	0.4686
Length	0.41	0.01	0.85	[0.40, 0.42]	4994	1	11543.8	< .001	0.1096
OLD20	– 0.77	0.01	– 0.56	[– 0.80, – 0.74]	952	1	2200.4	< .001	0.0209
Zipf * OLD20	0.22	0.01	0.53	[0.21, 0.23]	550	1	1271.8	< .001	0.0121
Zipf * length	– 0.07	0.002	– 0.61	[– 0.07, – 0.07]	522	1	1207.1	< .001	0.0115
Residuals					17,637	40,771			

SE = standard error. CI = confidence interval. SS = sums of squares. *df* = degrees of freedom. *η*^2^ = effect size (eta-squared)

#### Word frequency

Word prevalence is highly related with lexical frequency: the more frequent a word is in a language, the greater the knowledge of this word tends to be. Thus, it is not surprising that frequency explains 46.86% of the variance of word prevalence scores. In fact, the correlation between prevalence and Zipf is high (i.e., .67; see Table 5), and larger than that observed in Dutch (.35) and English (.49). It should be noted, however, that the differences in the approaches used in the three studies suggest caution in the comparison between them: While we included all the words in the correlation analysis, Brysbaert et al. (2019) did not consider all the frequency ranges, and Keuleers et al. (2015) only included a relatively small subset of words.

**Fig. 12:**
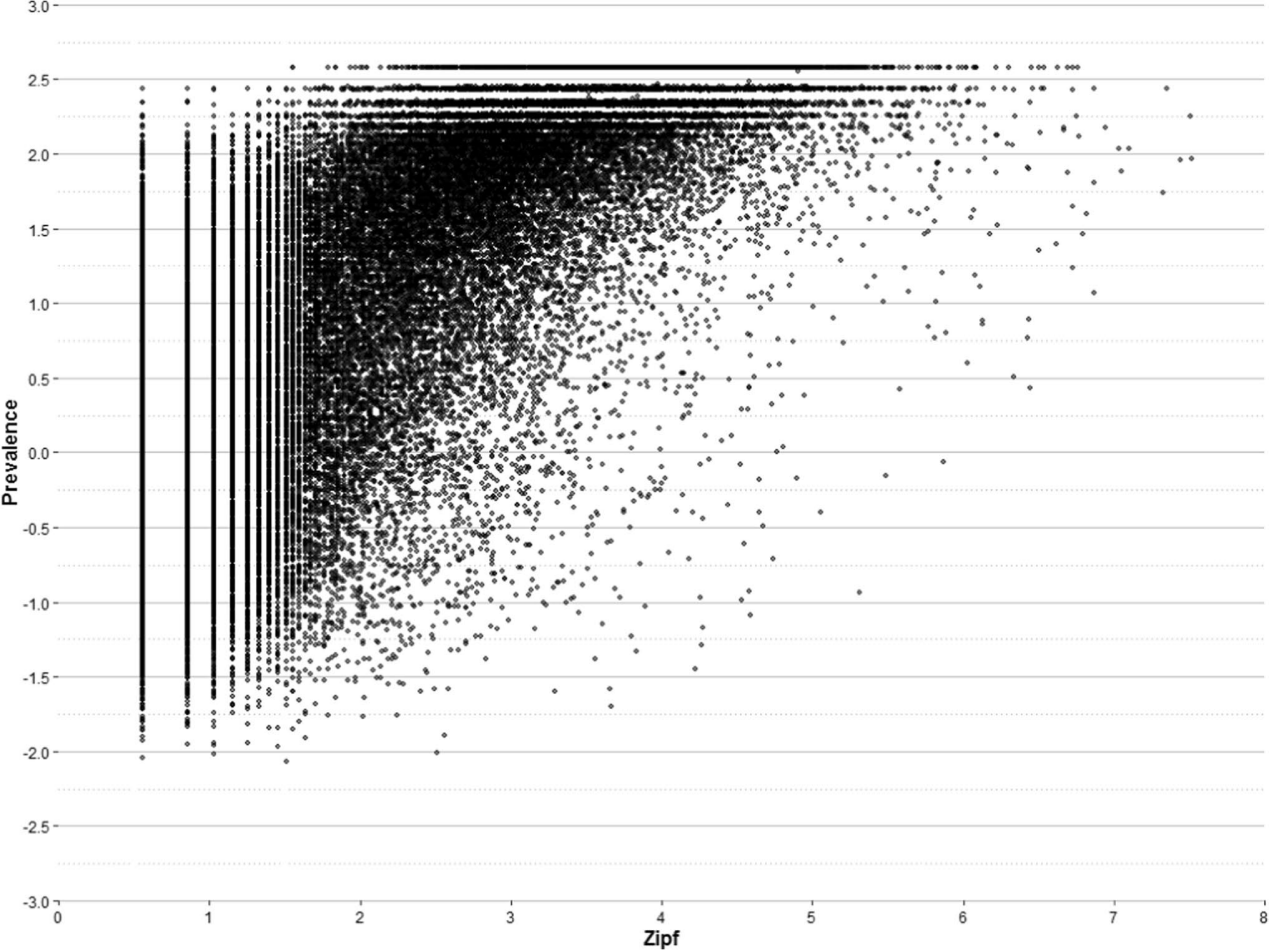
Scatterplot of the relationship between word frequency and prevalence

Despite the high correlation between frequency and prevalence, it would not be adequate to consider both variables as two ways of looking at the same phenomenon. In the seminal study on word prevalence, Keuleers et al. (2015) demonstrated a significant contribution of this variable in predicting reaction times in lexical decision tasks, with a unique contribution, which was independent of lexical frequency. To date, there is not a database of lexical decision times that allows to examine this issue in Catalan. However, we can gain an insight into the independence of the two variables by looking at Fig. 12.

As can be seen, word knowledge tends to increase as frequency does. However, a close inspection of Fig. 12 reveals two interesting patterns. Firstly, the vertical bars at the left side of the plot display words that, despite having a very low lexical frequency, differ in prevalence across the entire range. Indeed, it is possible to find very low frequency words that are known by almost all the population (e.g., reddish, seismography, or logarithmic; with a percentage of knowledge greater than 95%, but a Zipf of 0.56). In contrast, the opposite (i.e.: high-frequency words that are not known by a large part of the population), is not observed. And secondly, the horizontal lines on the top of the plot display words that, despite being known by a large part of the population, differ practically throughout the entire possible frequency range. This pattern is very similar to that previously described in Dutch (Keuleers et al., 2015).

#### Word length

**Fig. 13:**
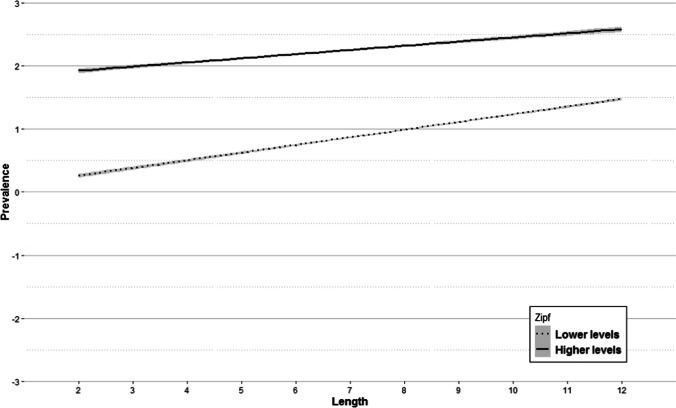
Regression lines of word length on prevalence divided by frequency: lower levels = *dotted line* and higher levels = *solid line*. The *shaded areas* denote a 95% confidence interval

This variable, with a correlation of .16 with word prevalence, explains 10.96% of the variance, placing it as the second predictor. In general, the longer the word, the more likely participants will know it. However, since the interaction between length and Zipf was significant (explaining a 1.15% of the variance), the effect of length should be examined in the context of this interaction. Figure 13 shows such interaction after dividing the observed range of Zipf values into two, taking the value of 4 as the split point. There were 92.1% of the words in the lower levels of Zipf, while the remaining 7.9% of the words were in the higher levels.

The interaction indicates that although length influences all frequency levels, its effect is greater for lower levels of frequency (dotted line) than for higher levels of frequency (solid line). In other words, the more frequent the words are, the lower the effect of length on the probability of knowing them. A similar interaction has been described in Spanish (Aguasvivas et al., 2020), with the same predictors over average accuracy per word.

#### Orthographic neighborhood

**Fig. 14:**
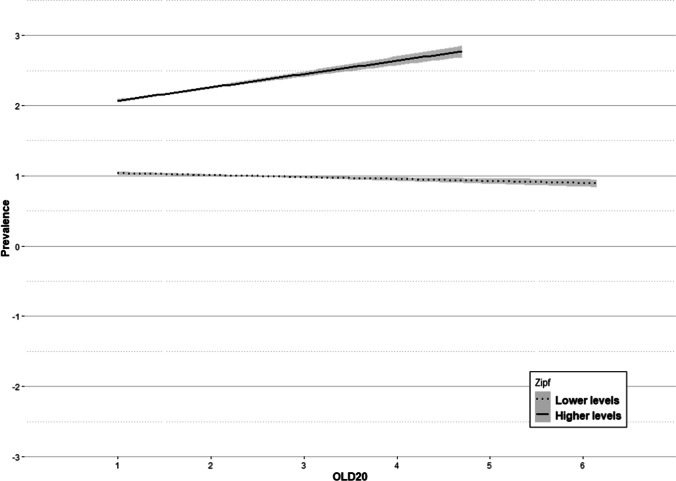
Regression lines of OLD20 on prevalence divided by frequency: lower levels = *dotted line* and higher levels = *solid line*. The *shaded areas* denote a 95% confidence interval

The density of the orthographic similarity neighborhood of a word explains a 2.09% of the variance, indicating that, in general, the sparser the neighborhood of a word is, the lower its prevalence. An additional 1.21% of variance is explained by the interaction of this variable with frequency (see Fig. 14).

In this case, and contrary to what happened with the interaction of frequency by length, the effect even reverses as frequency increases. Thus, the knowledge of low-frequency words slightly tends to decrease as the distance to the orthographic neighborhood increases. In contrast, words with higher levels of frequency are better known if they are more different from other words. This result contrasts with the one reported by Aguasvivas et al. (2020), who observed that a high OLD20 predicted a higher accuracy, and that this influence was higher for low-frequency words than for high-frequency words.

## Conclusions

In this work, we have followed the approach of the crowdsourced mega-studies that have so far examined word prevalence in visual word recognition as a complementary measure to lexical frequency. The data obtained with this type of studies are more representative of the word knowledge of the general population (i.e., there is more variability in the age and gender distribution of the participants, as well as in their education level) than those gathered in traditional laboratory studies, although still not representative of the total population.

In the present study, more than 200,000 native speakers of Catalan have participated, and the prevalence of more than 40,000 words has been estimated. Therefore, Catalan adds to Dutch, English, and Spanish, as the languages with data on word prevalence. The results of the analyses carried out are very similar to those reported in these languages. Regarding the properties of the items, most of the variance in word prevalence was explained by word frequency. Furthermore, both word length and orthographic neighborhood interacted with this variable. Concretely, while low-frequency words were the most affected by the facilitative effects of length on word knowledge, high-frequency words were the most affected by the facilitative effect of orthographic neighborhood.

Concerning the characteristics of the participants, vocabulary size was affected, in this order, by age, education level, number of spoken languages, and Catalan proficiency. Particularly, older participants, those who had a higher education level, those who spoke more languages, and those who rated themselves as more proficient in Catalan, were those who knew more Catalan words. A small effect of gender was observed, too, with a slight advantage for male participants. Further research is needed to elucidate the mechanisms of these effects. For instance, orthographic overlap between translation equivalents (i.e., cognate effect) has been proposed as explanation for the facilitative effect of number of languages (Aguasvivas et al., 2020; Keuleers et al., 2015). This might be tested by computing the percentage of cognate words between the target language of the mega-study and the other languages known by the speakers and examining if vocabulary size depends on that percentage. Similarly, the possible interactions between the assessed variables (e.g., gender and age, gender and education level) should be tested in future studies involving an even more varied population of speakers to establish their possible role in vocabulary size.

The results of the analyses carried out in this study as well as those of previous crowdsourced mega-studies suggest that word prevalence is a relevant measure to be considered in psycholinguistic research. Brysbaert et al. (2019) warned against the inclusion of words with very low prevalence in reaction time experiments, because these words are not known by most speakers. Furthermore, the experimental conditions should be matched not only in word frequency, but also in word prevalence, considering the role of this variable in visual word recognition (Brysbaert et al., 2016a; Brysbaert et al., 2019; Keuleers et al., 2015). Apart from that, word prevalence may have value in and of itself as an index of word difficulty. For instance, it can be used to assess the difficulty of written texts, to select words for word-learning experiments or for the development of standardized vocabulary tests among others (Brysbaert et al., 2019). In relation to that, a note of caution should be introduced here: Normative data about word prevalence have been obtained in lexical decision studies, and the predictive capacity of this variable on visual word recognition time has been tested gathering reaction times from other lexical decision studies (except for the word naming data analyzed by Brysbaert et al., 2019). Therefore, although the results to date are promising, more research is needed, using other tasks, to further validate word prevalence as a measure of word difficulty.

This database, together with the most recent database of lexical frequency in Catalan to date (SUBTLEX-CAT; Boada et al., 2020), provides the basic tools to carry out lexical recognition studies in this language. Catalan is a co-official language with Spanish in Catalonia, where there is a strong presence of both languages. Therefore, most inhabitants are highly proficient in Catalan and Spanish. Among this population, the word prevalence data presented here have been obtained from speakers who have Catalan as their native (dominant) language. However, a non-negligible proportion of the speakers have Spanish as their native (dominant) language. Those participants were not included in the analyses presented in this paper, because the word prevalence measure is commonly obtained from native speakers (Aguasvivas et al., 2020; Brysbaert et al., 2019; Keuleers et al., 2015). The comparison between these two groups of speakers (i.e., Catalan dominant bilinguals and Spanish dominant bilinguals) is relevant and will be the topic of further studies, as it has been done recently in other languages (Brysbaert et al., 2021). On the other hand, the existence of data about the prevalence of words that are used on a daily basis by the same speakers in two different languages (Catalan and Spanish) opens the door to future advances in the field of bilingualism and second language acquisition.

## Footnotes

1 Note that we could only know that more than one record belonged to the same participant if he/she used the option to participate once again in the same session without having to fill in the questionnaire for the second time (i.e., it was not possible to identify people who participated more than once on different devices or days; see the Procedure below).

2 To test the method developed to compensate for the possible unequal occurrence of items and to avoid large differences in the number of observations, 70 words and 30 nonwords were left out of it to check if the system worked. For this reason, this small group of items has around 2000 observations. If these items were not considered, the mean number of observations would be 418.34 (SD = 15.14; range [350–478]) for words, and 241.79 (SD = 15.98; range [182–304]) for nonwords.
